# Case Report: Cervical lymphadenitis resulting from *Pseudomonas aeruginosa* diagnosed by metagenomic next-generation sequencing

**DOI:** 10.3389/fped.2026.1795457

**Published:** 2026-05-18

**Authors:** Lihong Gao, Yang Wen, Xiaoyu Jing

**Affiliations:** 1 Department of Pediatric Infectious Diseases Nursing Unit, West China Second University Hospital, Sichuan University, Chengdu, Sichuan, China; 2 Key Laboratory of Birth Defects and Related Diseases of Women and Children (Sichuan University), Ministry of Education, Chengdu, Sichuan, China; 3 Department of Pediatric Infections Diseases, West China Second University Hospital, Sichuan University, Chengdu, Sichuan, China; 4 Department of Pediatric Hematology Diseases, West China Second University Hospital, Sichuan University, Chengdu, Sichuan, China

**Keywords:** case report, cervical lymphadenitis, metagenomic next-generation sequencing, Pseudomonas aeruginosa

## Abstract

Pediatric cervical lymphadenitis is usually caused by *Staphylococcus aureus* and *Streptococcus pyogenes*. Cases resulting from Gram-negative bacteria are rare. Herein, we report the case of an 11-year-old boy who developed cervical lymphadenitis. He was diagnosed with a *Pseudomonas aeruginosa* infection through metagenomics next-generation sequencing of blood and biopsy. After treatment with meropenem, the patient’s condition improved and he was discharged. Lymphadenitis may be caused by Gram-negative opportunistic pathogens. Metagenomic next-generation sequencing can help identify the underlying cause.

## 1 Introduction

Cervical lymphadenitis is prevalent in children. Lymphadenitis is suspected to result from an inflammatory process (1). *Staphylococcus aureus* (*S. aureus*) and *Streptococcus pyogenes* (*S. pyogenes*) are responsible for the majority of infections in children under 4 years (2). Anaerobic infections should be suspected in older children and adolescents, especially in the setting of poor dentition or periodontal disease (3).

Herein, we report the case of an 11-year-old boy with cervical lymphadenitis caused by *Pseudomonas aeruginosa* (*P. aeruginosa*). Metagenomic next-generation sequencing (mNGS) was performed to identify the pathogen. *P. aeruginosa* was detected in blood and biopsy tissue by mNGS, but the results of the bacterial culture tests of these samples were negative.

## 2 Case presentation

An 11-year-old boy was admitted to hospital because of a cervical mass that prevailed for 6 days. The patient developed a fever of 38 °C. On admission, a physical examination revealed a rubbery, fixed, solid mass with tenderness in the right neck, measuring 5 cm × 5 cm. The skin overlying the infected lymph node was warm and erythematous. The boy had no history of cat scratches or contact with patients suffering from tuberculosis.

The complete blood count results were all within normal limits. The rate of erythrocyte sedimentation was 96 mm/h, and the level of C-reactive protein was 40.3 mg/L. Screening test results for viral and tuberculosis infections were all negative. Blood culture results also yielded negative results. Additional laboratory investigations for immunodeficiency and other predisposing conditions were performed, which revealed serum immunoglobulin G (IgG), immunoglobulin A (IgA), and immunoglobulin M (IgM) levels within the normal range.

A radiologic report revealed multiple enlarged lymph node shadows in the right neck. The largest one was adjacent to the right parotid gland, approximately 1.9 cm × 1.9 cm in size, and the swollen lymph nodes coalesced into a mass. Empiric oxacillin against *S. aureus* and *S. pyogenes* was administered. However, the patient remained febrile, with progressive worsening of the swelling after 4 days of treatment. Lymphadenitis caused by methicillin-resistant *S. aureus* (MRSA), anaerobic bacteria, or Gram-negative pathogens was suspected. Vancomycin, ceftriaxone, and metronidazole were given to target these pathogens.

Concurrently with antimicrobial therapy, immunological investigations were carried out. The boy’s cellular immunity was basically normal, with only the CD4/CD8 ratio being 0.9 (reference range: 1.5–2.0). His human immunodeficiency virus (HIV) test result was negative. The boy’s guardians were informed about the need to perform a local tissue biopsy. Weighing the procedural discomfort against the diagnostic yield, they opted for postponement of the intervention and continuation of conservative anti-infective management with close clinical monitoring.

However, the patient’s condition deteriorated after 8 days, with a fever reaching 40℃ and palpable swollen lymph nodes on the other side of the neck. There was a high suspicion of non-infectious diseases, especially lymphoma. A lymph node dissection and biopsy were performed on the patient. However, the preliminary pathological findings did not indicate neoplastic disease. mNGS was introduced to identify the pathogens from the biopsy and blood specimens. Simultaneous test results, including those of aerobic and anaerobic bacterial cultures, fungus culture, and mycobacterial culture using the same biopsy specimen, were all negative. Positron emission tomography–computed tomography (PET-CT) revealed that the swollen cervical lymph nodes were consistent with inflammatory disease. Then, the pathological examination and immunohistochemical test results were returned and revealed the diagnosis of benign lymphoproliferative disease. The result of mNGS in the lymph node biopsy specimen showed two reads corresponding to the bacterium genome, with a coverage rate of 0.0024%. *P. aeruginosa* was also detected in the peripheral blood by mNGS. A diagnosis of cervical lymphadenitis secondary to *P. aeruginosa* infection was favored. After the boy was treated with meropenem for 3 days, his body temperature gradually returned to normal. The favorable clinical response validated the efficacy of the ongoing antimicrobial therapy. The cervical mass almost disappeared after two weeks. The genes of immunodeficiency were detected, and no positive evidence was found. The boy was discharged from the hospital without any symptoms. The detailed diagnostic and therapeutic course is provided in Table 1.

**Table 1:** Diagnosis and treatment process.

Hospital day	Key events(symptoms/examinations)	Key diagnostic findings	Key treatments
Day 1–5 (outpatient department)	Cervical mass	None	Without treatment
Hospital Day 1–3	Fever; a rubbery, fixed, solid mass with tenderness on the right neck sites; multiple enlarged lymph nodes shadowed in the right neck	The results of the complete blood count were normal. The erythrocyte sedimentation rate was 96 mm/h, and the C-reactive protein level was 40.3 mg/L. The results of virus and tuberculosis infection screening tests were all negative.	Intravenous empirical oxacillin
Hospital Day 3 and 4	Fever; cervical mass remains unchanged	Blood culture tests showed negative results. Serum IgG, serum IgA, and serum IgM levels were within the normal range.	Intravenous empirical oxacillin
Hospital Day 5–12	Persistent fever and worsening swelling	It was suspected that lymphadenitis might be caused by methicillin-resistant *S. aureus* (MRSA), anaerobic bacteria, or Gram-negative pathogens.	Intravenous vancomycin, ceftriaxone, and metronidazole (for 8 days)
Hospital Day 13 and 14	Fever reaching 40 ℃ and palpable swollen lymph nodes on the other side of the neck	A lymph node dissection and biopsy did not indicate neoplastic disease. The result of mNGS in the lymph node biopsy specimen showed two reads corresponding to the bacterium genome. The results of aerobic and anaerobic bacterial culture tests, fungus culture test, and mycobacterial culture test using the same biopsy specimen were all negative. *P. aeruginosa* was detected in the peripheral blood by mNGS. PET-CT suggested inflammatory disease.	Intravenous meropenem
Hospital Day 15–25	Normal body temperature	No new diagnostic findings	Intravenous meropenem (for 2 weeks)
Hospital Day 26	The cervical mass disappeared.	Pathological test and immunohistochemical test results revealed a diagnosis of benign lymphoproliferative disease; there were no gene abnormalities in immunodeficiency.	Improvement in the patient’s condition and discharge

## 3 Discussion

Pediatric cervical lymphadenitis is common. It can present with various symptoms, including lymph node enlargement, fever, malaise, erythema, edema, and pain in the overlying skin. Pediatric unilateral cervical lymphadenitis is typically caused by an infectious agent, with *S. aureus* and *S. pyogenes* being the most common bacterial pathogens. For patients with concurrent dental or periodontal disease, anaerobic bacteria should be the suspected cause (4). The boy had poor oral hygiene. Although there were no dental or perioral diseases, anaerobic infection remained a diagnostic consideration, which prompted the addition of metronidazole to the therapeutic regimen. Moreover, MRSA has become a common pathogen of skin and soft tissue infections and should be considered in cases of cervical lymphadenitis (2, 4). For patients who have an inadequate response to 48–72 h of antimicrobial therapy or those with physical findings of localized fluctuance or swelling, needle aspiration of lymph nodes is an effective initial intervention (1). In the case of this patient, lymph node dissection and biopsy were performed on day 8 of treatment because of the persistently suboptimal clinical response, in order to further elucidate the underlying etiology.

Pediatric cervical lymphadenitis caused by Gram-negative bacteria is rare. *P. aeruginosa* is a Gram-negative opportunistic bacterium, which is part of the normal intestinal flora and exists in hospital infrastructure such as respiratory equipment and dialysis tubing (5). It poses a significant threat to patients who are immunocompromised, causing infections that range from acute to chronic (6). Traditional culture methods are the gold standard for detecting *P. aeruginosa*, but they usually yield false-negative results because of the low load of pathogenic bacteria and previous antibiotic use (7). In addition, the use of broad-spectrum antibiotics for unclear infections increases the resistance of *P. aeruginosa*, making its treatment more difficult (8).

Only two similar cases had been published previously: the case of a 15-year-old patient with *P. aeruginosa* cervical lymphadenitis in 1969 (in Slovak) and that of a 13-month-old male baby with *P. aeruginosa* axillary lymphadenitis in 2008 (9, 10). *P. aeruginosa* is a prevalent opportunistic human pathogen and usually causes hospital-acquired infections in immunocompromised individuals. Infection by *P. aeruginosa* in patients with immunocompetent is rare. However, our patient did not have any apparent predisposing condition, and neither he nor his guardians were able to provide a reliable history regarding potential exposure to contaminated water sources. After the boy was infected with *P. aeruginosa*, the body's immune system was activated. The persistent immune stimulation may lead to reactive lymph node enlargement and lymphocytic hyperplasia, ultimately resulting in benign lymphoproliferative disease.

mNGS, as a rapidly developing molecular biology technology, has been used to rapidly detect infectious pathogens. mNGS shows significantly higher positive rates and timeliness for pathogen detection than traditional culture-based diagnostics (11). In addition, mNGS can provide more genetic information, including evolutionary tracing, strain identification, and prediction of drug resistance. However, because of the high sensitivity of mNGS, background microbes may be identified as causative pathogens, resulting in false-positive results. To date, there is no standard for verifying whether the microorganisms detected by mNGS are pathogens or background microorganisms. However, this can be indirectly verified by whether the antibiotic treatment selected based on the mNGS results is effective. In the case of the patient in this study, only two reads of *P. aeruginosa* were revealed by mNGS. This test did not set a negative control, and therefore, the possibility of background bacteria or contamination cannot be completely ruled out. However, *P. aeruginosa* was detected in the biopsy specimen and blood. The clinical symptoms of this patient alleviated after the use of meropenem. Therefore, based on the concurrent detection of *P. aeruginosa* in both biopsy specimens and blood samples, along with the observed treatment response, the patient was ultimately diagnosed with cervical lymphadenitis caused by *P. aeruginosa*.

## 4 Conclusion

*P. aeruginosa* can cause cervical lymphadenitis in children. When conventional treatments are ineffective, the possibility of infection by this bacterium should be considered. mNGS is a reliable diagnostic tool that can help clinicians identify the pathogen in different types of samples.

## Data Availability

The original contributions presented in the study are included in the article/supplementary material, further inquiries can be directed to the corresponding author/s.
